# Supplementary material for: Soft, tough, and fast polyacrylate dielectric elastomer for non-magnetic motor
Source: Nat Commun. 2021 Jul 26;12:4517. doi: 10.1038/s41467-021-24851-w (PMC8313586; doi:10.1038/s41467-021-24851-w)
Supplement: Supplementary file 1 — Supplementary Information [file 41467_2021_24851_MOESM1_ESM.pdf]

Supplementary Information

Soft, tough, and fast polyacrylate dielectric elastomer for non-magnetic motor

Li-Juan Yin[1], Yu Zhao[2], Jing Zhu[1], Minhao Yang[3], Huichan Zhao[4*], Jia-Yao Pei[1], Shao-Long Zhong[1] & Zhi-Min Dang[1,2*]

[1] State Key Laboratory of Power System, Department of Electrical Engineering, Tsinghua University, Beijing, 100084, China

[2] School of Electrical Engineering, Zhengzhou University, Zhengzhou, 450001, China

[3] Institute of Advanced Materials, North China Electric Power University, Beijing, 102206, China

[4] Department of Mechanical Engineering, Tsinghua University, Beijing 100084, China

*e-mail: zhaohuichan@tsinghua.edu.cn; dangzm@tsinghua.edu.cn

Supplementary Discussion

Toughness. Apart from enhancing dielectric constant, lowering the elastic modulus is another feasible approach for the increase of the actuation sensitivity of elastomer films. However, materials with low moduli are usually delicate, consequently restricting their applications[1-4]. Thus, toughness (K), discussed here, is defined as the area surrounded by the stress (σ) - strain (λ) curves from uniaxial stretch using equation[5]:

$$K(\mathrm{MJ\ m^{-3}}) = \int_{\lambda=0}^{\lambda=\lambda_{\max}} \sigma d\lambda$$

where, σ is the nominal stress (MPa) and λ is the strain (mm mm^{-1}).

Compared with the pervious DEs with a low elastic modulus (< 1 MPa), polyacrylate dielectric elastomers synthesized in this work exhibits an overwhelming advantage on toughness (Fig. 1e and Supplementary Fig. 4). Despite owning an ultralow Young's modulus, BAC2 also has excellent stretchability and tensile strength due to the rationally crosslinked network and the existence of trace amounts of hydrogen bonds[6]. The combination of low modulus and high toughness makes the synthesized elastomer desirable in the different applications.

Calculation of transient time constant and simulation of electrically driving frequency. The theoretical analysis of the charging process was carried out through an equivalent circuit model. Supplementary Fig. 11a displays the schematic of the equivalent circuit model of non-magnetic motor. Since the electromechanical response speed of DEAs not only depends on mechanical properties like viscoelasticity and stiffness but also is related to electrical parameters. The evolution of voltage applied to elastomer film with the time during the charging process will be systematically

discussed as follows.

When a fixed bias voltage is applied between two electrodes, there is a transient process for voltage establishment on elastomer film.

According to Kirchhoff's voltage law,

$$U_{\mathrm{s}} = (R_1 + R_{\mathrm{s}})i + U_{\mathrm{c}} \qquad (1)$$

where R_1 is the protective resistance, R_{s} is the equivalent resistance of electrodes, i is the output current of power supply, and U_{s} and U_{c} are the voltages of power supply and capacitor, respectively.

Next, according to Kirchhoff's current law,

$$i = C_{\mathrm{p}}\frac{\mathrm{d}U_{\mathrm{c}}}{\mathrm{d}t} + U_{\mathrm{c}}\frac{\mathrm{d}C_{\mathrm{p}}}{\mathrm{d}t} + \frac{U_{\mathrm{c}}}{R_{\mathrm{p}}} \qquad (2)$$

where R_{p} and C_{p} are the resistance and capacitance of the equivalent parallel model of elastomer film as a plate capacitor. Since mechanical response is much slower than electrical response, the capacitance change of C_{p} could be neglected. Thus, the following equation can be derived from Supplementary Equation (1) and (2).

$$\frac{(R_1+R_{\mathrm{s}})R_{\mathrm{p}}C_{\mathrm{p}}}{R_1+R_{\mathrm{s}}+R_{\mathrm{p}}}U_{\mathrm{c}}' + U_{\mathrm{c}} = \frac{R_{\mathrm{p}}}{R_1+R_{\mathrm{s}}+R_{\mathrm{p}}}U_{\mathrm{s}} \quad (3)$$

It's easy to obtain the solution of Differential Equation of the first-order.

$$U_{\mathrm{c}} = \frac{R_{\mathrm{p}}}{R_1+R_{\mathrm{s}}+R_{\mathrm{p}}}U_{\mathrm{s}}\left[1 - e^{-\frac{R_1+R_{\mathrm{s}}+R_{\mathrm{p}}}{(R_1+R_{\mathrm{s}})R_{\mathrm{p}}C_{\mathrm{p}}}t}\right] \quad (4)$$

Time constant (τ) can be gotten from Supplementary Equation (4).

$$\tau = \frac{(R_1+R_{\mathrm{s}})R_{\mathrm{p}}C_{\mathrm{p}}}{R_1+R_{\mathrm{s}}+R_{\mathrm{p}}} = C_{\mathrm{p}}\left[R_{\mathrm{p}}//(R_1 + R_{\mathrm{s}})\right] \quad (5)$$

$R_{\mathrm{p}} = \frac{\rho d}{A}$ and $C_{\mathrm{p}} = \frac{\varepsilon_0\varepsilon_{\mathrm{r}}A}{d}$, where ρ, ε_0 and ε_{r} are volume resistivity, permittivity of vacuum and relative dielectric constant, respectively. A and d are the surface area and thickness of film after stretched, respectively.

From the engineering point of view, it usually needs 3τ~5τ for the voltage to achieve the steady-state value $\frac{R_p}{R_1+R_s+R_p}U_s$ (Supplementary Equation (4)). Therefore, the driving frequency is limited to such a narrow range $(1 * \alpha/(3\tau \sim 5\tau))$, where α is the duty ratio of square wave voltage. As shown in Supplementary Fig. 11a, the frequency limitation is set to 500 Hz by adjusting the parameters of circuit elements. When the power frequency is well below 500 Hz, for example 50 Hz, the voltage on capacitor can reach the steady-state value (Supplementary Fig. 11b, c). On the contrary, when the power frequency exceeds 500 Hz, for example 5000 Hz, the voltage on capacitor is much lower than the steady-state value due to unfinished charging process within extremely short time (Supplementary Fig. 11d).

As for the non-magnetic motor, a thorough analysis and simulation were conducted. The high-field volume resistivity of BAC2 and VHB™ 4910 were measured by two-probe method. The volume resistivity of BAC2 is of the order of magnitude of 10^{11} Ω m, which is two orders of magnitude smaller than that of VHB™ 4910. And the relative dielectric constant of BAC2 and VHB™ 4910 is around 5.75 and 4.4 at 1 kHz, respectively. After equiaxially stretched to 4×4 times, the film was cut into a circle with a radius of 70 mm and thickness of 62.5 μm. Therefore, for BAC2, C_p is 11.5 nF and R_p is 4.43×10^8 Ω. For VHB™ 4910, C_p is 8.8 nF and R_p is 2.2×10^{11} Ω.

However,

$$R_1 + R_s = \frac{U_s}{i(0_+)} \qquad (6)$$

where U_s is of the order of the magnitude of kV and $i(0_+)$ is less than 1 mA. In fact, the resistance of $R_1 + R_s$ is of the order of the magnitude of 10^6 Ω, which is far less

than the resistance of R_{p}.

Therefore, Supplementary Equation (5) can be simplified into the following one,

$$\tau \cong C_{\mathrm{p}}(R_1 + R_{\mathrm{s}}) \qquad (7)$$

and τ is in the magnitude of 10^{-2} s.

Actually, the discussion above is greatly simplified. Supposing the capacitance is a constant value during actuation, we simulated voltage on film and current from power supply (Supplementary Fig. 12). The obtained results could be concluded as follows: (i) Current did not pass the limitation (1 mA) during the whole simulation; (ii) When driving frequency exceeded about 3 Hz, voltage on film of both VHB™ 4910 and BAC2 went down. As the driving frequency range of BAC2-based motor is much larger than 3 Hz, upper limit of rotation speed not only was determined by BAC2 elastomer itself but also was related to experimental device.

Supplementary Table 1 Comparison of mechanical properties among acrylic dielectric elastomers

	Young's modulus (MPa)	Elongation ($\lambda = L/L_0$)	Ultimate true strength (MPa)	Ultimate true strength/ Young's modulus	Toughness ($\mathrm{MJ\ m^{-3}}$)	Density ($\mathrm{kg\ m^{-3}}$)	Estimated $\overline{M_{\mathrm{c}}}$* ($\mathrm{g\ mol^{-1}}$)	$\overline{M_{\mathrm{n}}}$** of crosslinker ($\mathrm{g\ mol^{-1}}$)
VHB™4910	0.211	15.16	14.20	64.57	4.37	1037	35917	--
BA-S	0.112	12.37	7.93	105.72	2.53	1122	73210	575
BA-M	0.147	9.73	9.14	96.21	3.12	1117	55531	$1.6 * 10^3$
BA-L	0.161	9.745	10.39	129.86	3.44	1061	48160	$6.8 * 10^3$
BAC2	0.073	23.97	32.24	366.34	6.77	1008	100911	$2.8 * 10^4$

*: $\overline{M_{\mathrm{c}}}$ means the average molecular weight among crosslinking points, and is estimated from the equation: $\overline{M_{\mathrm{c}}} = 3\rho RT/Y$, where ρ, R, T and Y are density of elastomers, ideal gas constant ($8.314\ \mathrm{J\ mol^{-1}K^{-1}}$), Kelvin temperature (293 K) and

Young's modulus.

**: Number-average molecular weight of crosslinker (polyethylene glycol diacrylate for BA-S; oligomer CN9893NS for BA-M; oligomer CN9014NS for BA-L and CN9021NS for BAC2). The data is obtained from GPC.

Supplementary Table 2 Formulation of acrylic dielectric elastomers

sample	nBA (wt%)	CN9021NS (wt%)	HMPP (wt%)
BAC1	100	30	1
BAC2	100	35	1
BAC3	100	40	1
BAC4	100	45	1

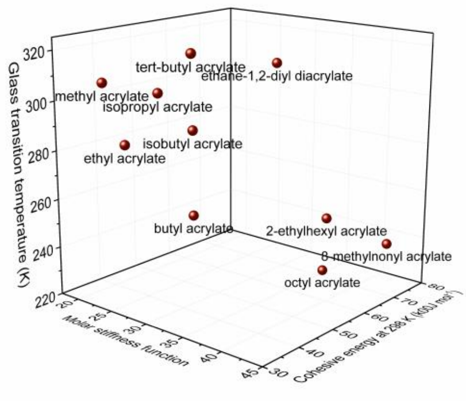

Supplementary Fig. 1 Comparison of molar stiffness function (MSF), cohesive energy (CE) and glass transition temperature (T_g) of homopolymers of commonly used acrylic monomers.

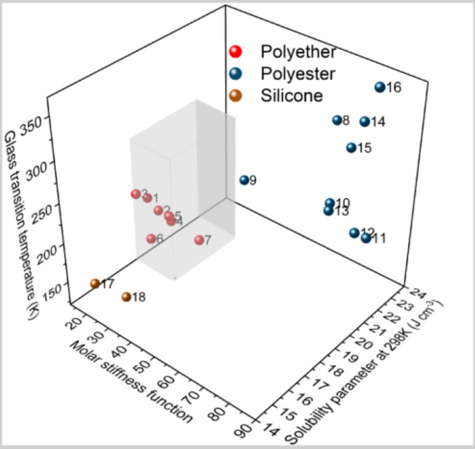

1 poly(ethane-1,2-diol)
2 poly(propane-1,3-diol)
3 poly(propane-1,2-diol)
4 poly(tetrahydrofuran)
5 poly(2-methylbutane-1,4-diol)
6 poly(2,2-dimethylpropane-1,3-diol)
7 poly(hexane-1,6-diol)
8 poly(2-(2-hydroxyethoxy)-2-oxoacetic acid)
9 poly(5-hydroxypentanoic acid)
10 poly(6-(2-hydroxyethoxy)-6-oxohexanoic acid)
11 poly(6-(4-hydroxybutoxy)-6-oxohexanoic acid)
12 poly(6-(2-(2-hydroxyethoxy)ethoxy)-6-oxohexanoic acid)
13 poly(6-(2-hydroxypropoxy)-6-oxohexanoic acid)
14 poly(3-((2-hydroxyethoxy)carbonyl)benzoic acid)
15 poly(2-((2-hydroxyethoxy)carbonyl)benzoic acid)
16 poly(4-((2-hydroxyethoxy)carbonyl)benzoic acid)
17 polydimethylsiloxane
18 polydiethylsiloxane

Supplementary Fig. 2 Comparison of MSF, T_g and solubility parameter (δ) of common flexible long chain oligomers containing polyester, polyether and silicone as repeat units.

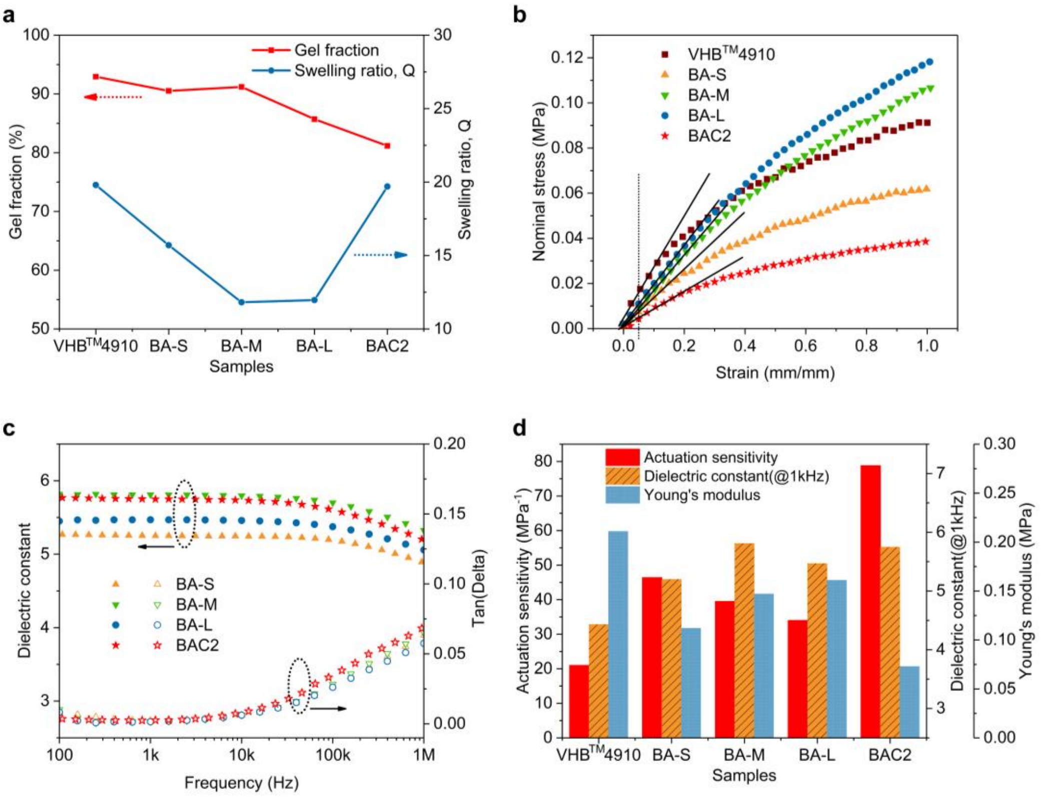

Supplementary Fig. 3 Dielectric and mechanical properties of VHB™4910 and BA-S, BA-M, BA-L, BAC2 samples. a, Gel test for VHB™4910, BA-S, BA-M, BA-L, and BAC2 samples. **b**, Stress-strain curves of VHB™4910, BA-S, BA-M, BA-L, and BAC2 samples. Young's moduli are defined as the slopes of tangent lines at 5% strain on stress-strain curves. **c**, Frequency dependence of dielectric constant and dissipation factor (Tan δ_e) of BA-S, BA-M, BA-L, and BAC2 samples. **d**, Comparison of actuation sensitivity, dielectric constant and Young's modulus of VHB™4910, BA-S, BA-M, BA-L, and BAC2 samples.

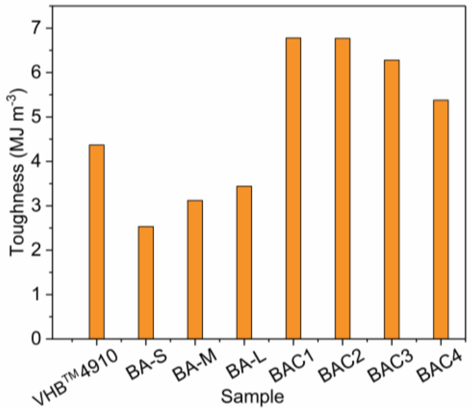

Supplementary Fig. 4 Comparison of toughness among VHB^{TM} 4910 and new polyacrylates.

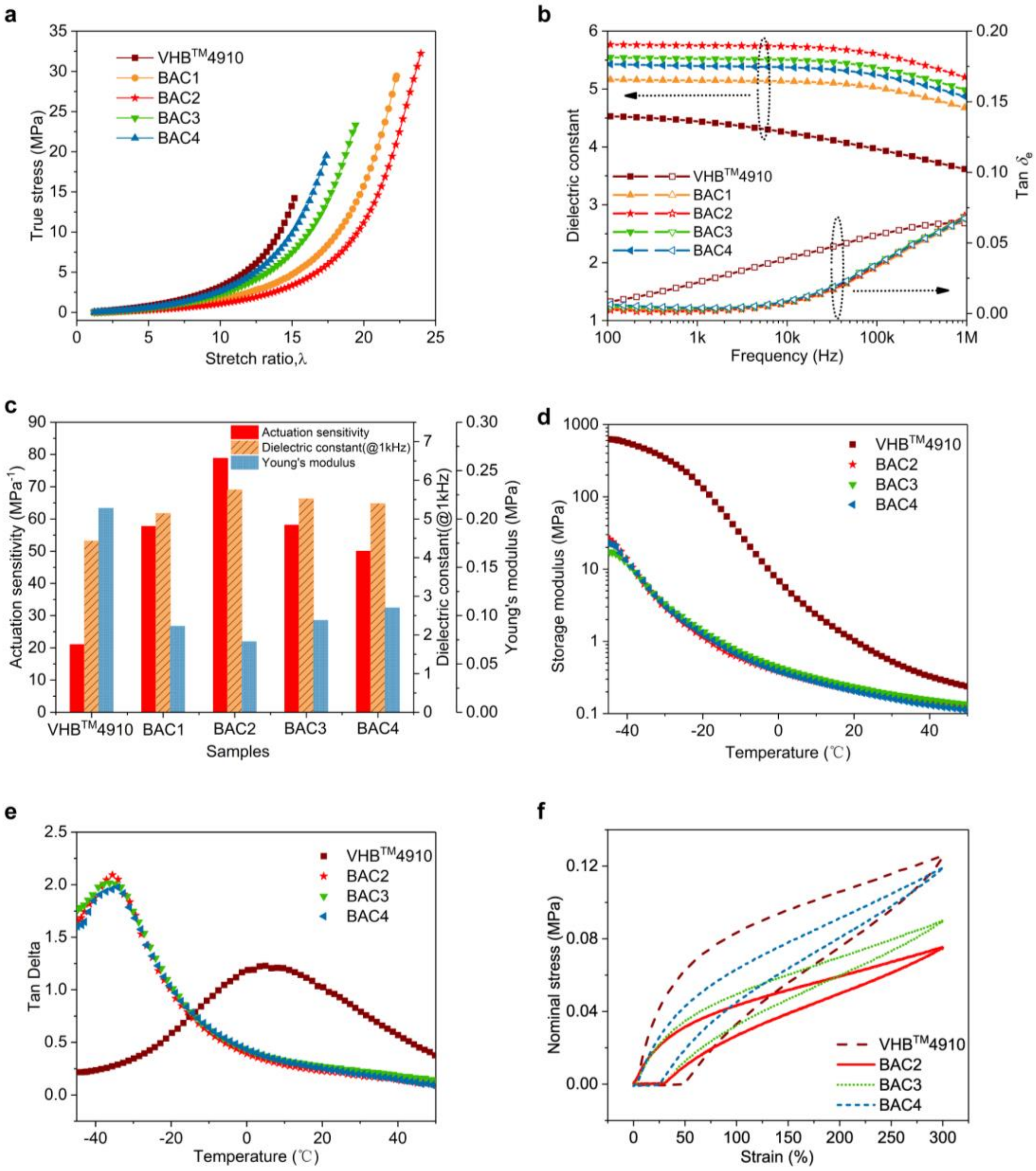

Supplementary Fig. 5 Dielectric and mechanical properties of VHB^{TM}4910 and BAC series samples. a, Stress-strain curves of VHB^{TM}4910, and BAC series samples. **b**, Frequency dependence of dielectric constant and dissipation factor (Tan δ_e) of VHB^{TM}4910 and BAC series samples. **c**, Comparison of actuation sensitivity, dielectric constant and Young's modulus of VHB^{TM}4910 and BAC series samples. BAC1 will also be ignored due to its sticky feature in the following characterizations. **d**, **e**, Temperature dependence of storage modulus (**d**) and mechanical loss (Tan δ_m) (**e**). **f**, Cyclic test of VHB^{TM}4910 and BAC series samples under uniaxial stretching.

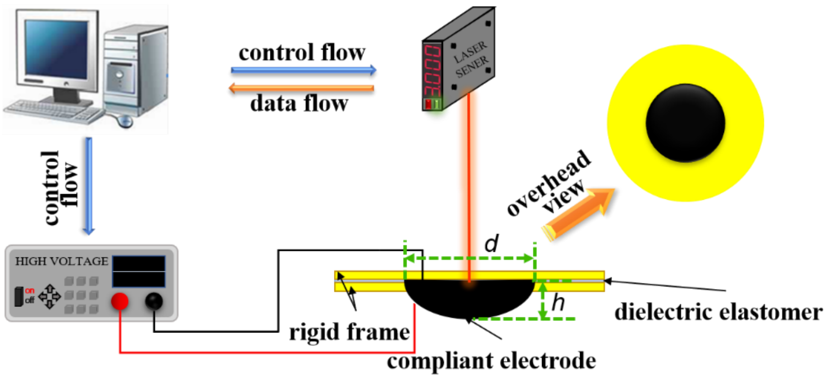

Supplementary Fig. 6 Schematic diagram showing the actuation performance test platform for elastomers without pre-strain.

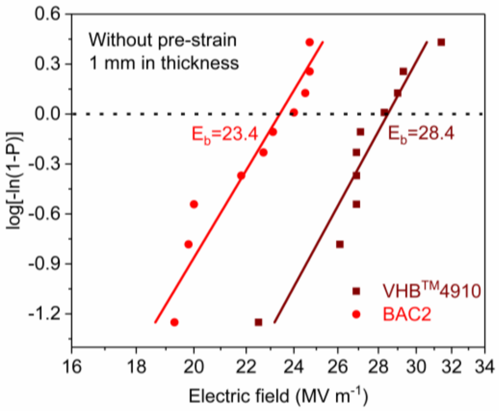

Supplementary Fig. 7 Comparison of electrical breakdown strength between VHB^{TM}4910 and BAC2 synthesized in this work.

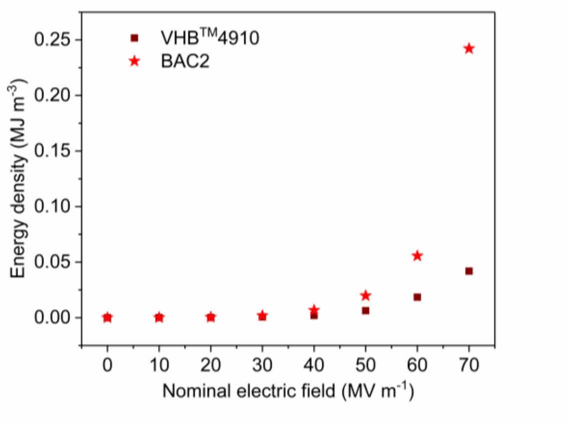

Supplementary Fig. 8 Dependence of estimated energy density on nominal electric field for VHB^{TM}4910 and BAC2.

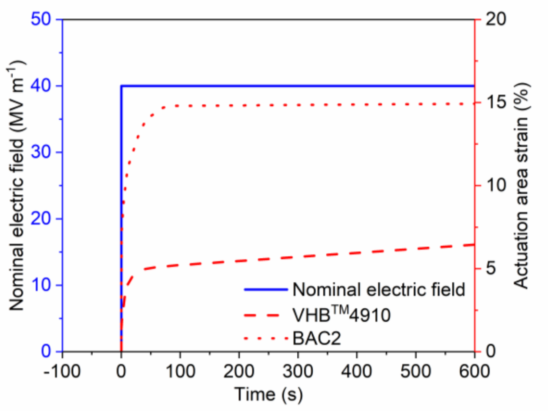

Supplementary Fig. 9 Time dependent behavior of actuation area strain of VHB^{TM} 4910 and BAC2 with 4×4 equiaxial pre-strain at 40 MV m^{-1}.

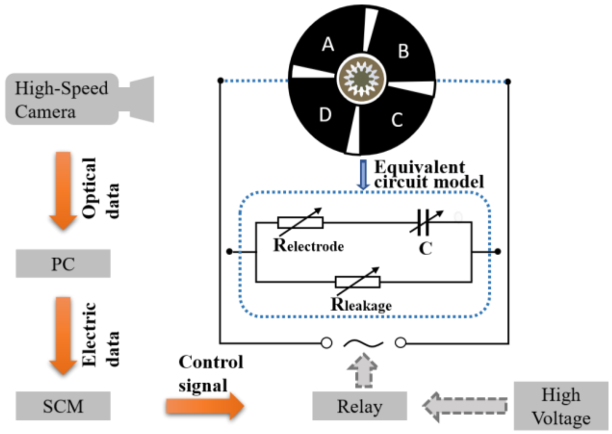

Supplementary Fig. 10 The schematic diagram of non-magnetic motor driving system.

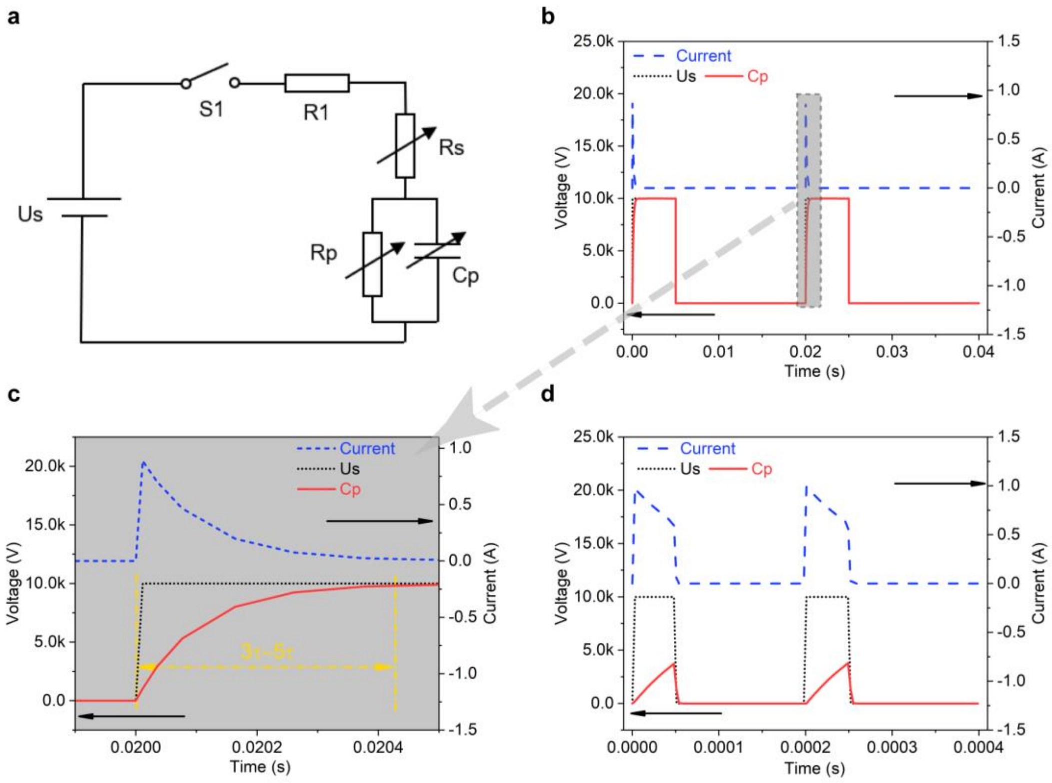

Supplementary Fig. 11 Theoretical analysis of the frequency-dependent behavior of the charging process for a simplified capacitor. a, Schematic of the equivalent circuit model of non-magnetic motor, among which U_s, S_1, R_1, R_s, R_p and C_p stands for the power supply during charging process, relay, protective resistor in series, total resistor of wires and electrodes, and resistance and capacitance of the equivalent parallel model of elastomer film as a plate capacitor, respectively. R_s, R_p and C_p are variable values since the dimension of elastomer film changes when an electric field is applied. **b**, Evolutions of loop current and voltage on C_p under the actuation of a periodic square wave voltage U_s at 50 Hz calculated by the Matlab/Simulink. **c**, The enlarged curves of content encircled by dashed line in Supplementary Fig. 11b. τ is the time constant for a transient process. **d**, Evolutions of current and voltage when the frequency is increased to 5000 Hz.

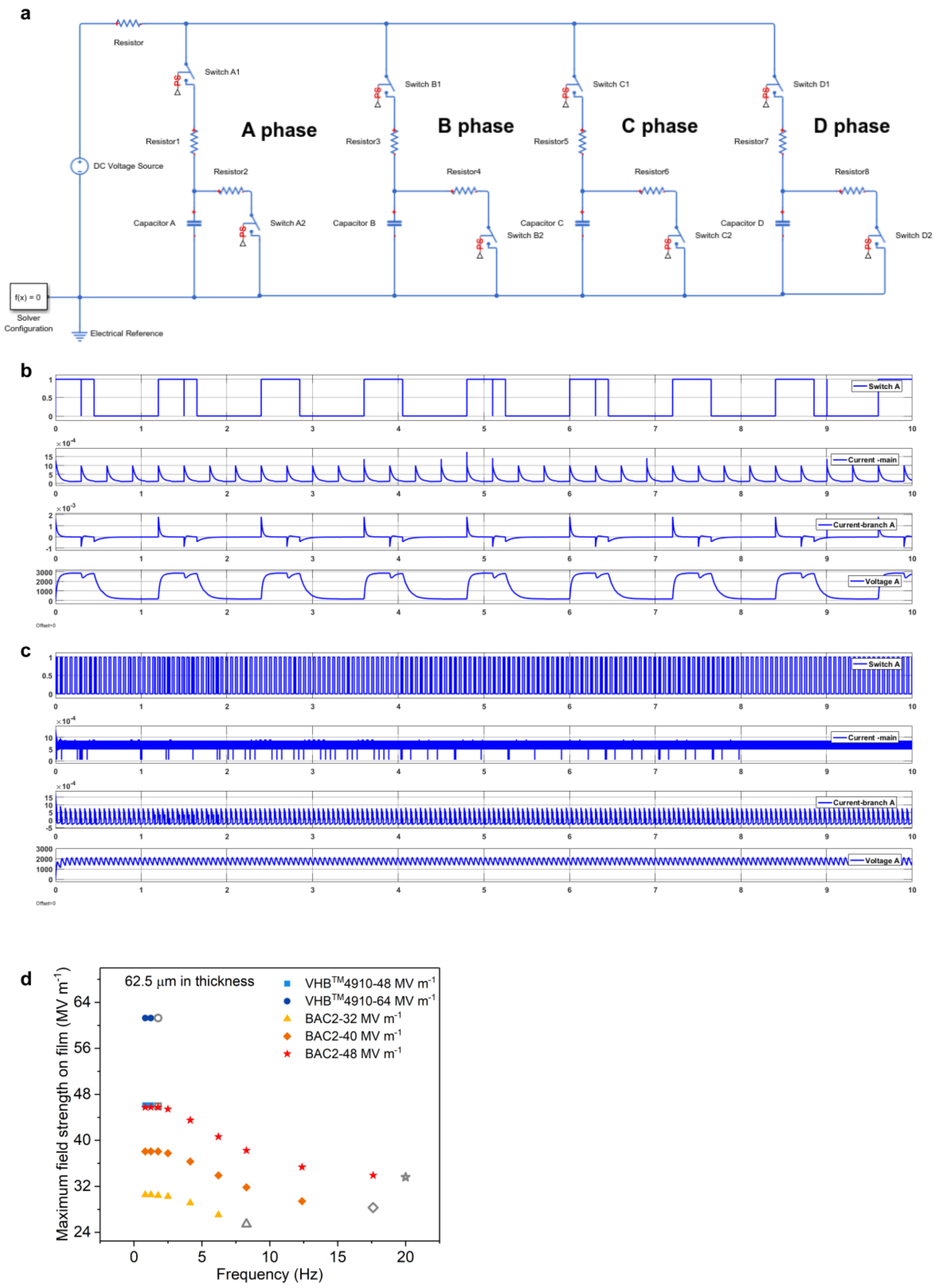

Supplementary Fig. 12 Simulation of soft motor. a, Equivalent circuit of soft motor. Control signal for switches (Relays) was not displayed here. **b**, At 0.833 Hz and 48 MV m^{-1} (3.2 kV), from top to down, these curves represent on-off state of Switch A1, current

from power supply, current through Switch A1 and voltage on phase A part of BAC2. **c**, At 17.61 Hz and 48 MV m^{-1} (3.2 kV), these curves represent on-off state of Switch A1, current form power supply, current through Switch A1 and voltage on phase A part of BAC2. **d**, Frequency dependence of maximum field strength on films of VHB^{TM}4910 and BAC2. Grey points denote the frequency where soft motor did not rotate.

Supplementary References

1. Chen, Z. et al. Ultrasoft-yet-strong pentablock copolymer as dielectric elastomer highly responsive to low voltage. *Chem. Eng. J.* **405**, 126634 (2021).
2. Shi, L. et al. Dielectric gels with ultra-high dielectric constant, low elastic modulus, and excellent transparency. *NPG Asia Mater.* **10**, 821-826 (2018).
3. Sheima, Y., Caspari, P. & Opris, D. M. Artificial Muscles: Dielectric Elastomers Responsive to Low Voltages. *Macromol. Rapid Commun.* **40**, 1900205 (2019).
4. Wallin, T. J. et al. 3D printable tough silicone double networks. *Nat. Commun.* **11**, 4000 (2020).
5. Liu, T. et al. Super-strong and tough poly(vinyl alcohol)/poly(acrylic acid) hydrogels reinforced by hydrogen bonding. *J. Mater. Chem. B*, **6**, 8105-8114 (2018).
6. Kang, J. et al. Tough and Water-Insensitive Self-Healing Elastomer for Robust Electronic Skin. *Adv. Mater.* **30**, 1706846 (2018).
