# Supplementary material for: Soft, tough, and fast polyacrylate dielectric elastomer for non-magnetic motor
Source: Nat Commun. 2021 Jul 26;12:4517. doi: 10.1038/s41467-021-24851-w (PMC8313586; doi:10.1038/s41467-021-24851-w)
Supplement: Supplementary file 3 — Description of Additional Supplementary Files [file 41467_2021_24851_MOESM3_ESM.pdf]

Description of Additional Supplementary Files

File Name: Supplementary Movie 1
Description: VHB™4910-based non-magnetic motor at different driving frequency under 48 MV m^{-1}

File Name: Supplementary Movie 2
Description: BAC2-based non-magnetic motor at different driving frequency under 48 MV m^{-1}

File Name: Supplementary Movie 3
Description: BAC2-based non-magnetic motor with transmission gears at different driving frequency under 48 MV m^{-1}

File Name: Supplementary Movie 4
Description: Process of pre-stretching using a custom-built rig
